# A novel *CISD2* mutation associated with a classical Wolfram syndrome phenotype alters Ca^2+^ homeostasis and ER-mitochondria interactions

**DOI:** 10.1093/hmg/ddx060

**Published:** 2017-03-06

**Authors:** Cécile Rouzier, David Moore, Cécile Delorme, Sandra Lacas-Gervais, Samira Ait-El-Mkadem, Konstantina Fragaki, Florence Burté, Valérie Serre, Sylvie Bannwarth, Annabelle Chaussenot, Martin Catala, Patrick Yu-Wai-Man, Véronique Paquis-Flucklinger

**Affiliations:** 1 Université Côte d’Azur, CHU, Inserm, CNRS, IRCAN, France; 2 Wellcome Trust Centre for Mitochondrial Research, Institute of Genetic Medicine, International Centre for Life, Newcastle University, Newcastle upon Tyne NE1 3BZ, UK; 3 Fédération de Neurologie, Université Pierre et Marie Curie et Groupe Hospitalier Pitié-Salpêtrière, Paris, France; 4 Joint Centre for Applied Electron Microscopy, Nice Sophia-Antipolis University, Nice, France; 5 UMR7592 CNRS, Jacques Monod Institute, Paris Diderot University, Paris, France; 6 UMR 7622 CNRS et UPMC et Fédération de Neurologie, Université Pierre et Marie Curie et Groupe Hospitalier Pitié-Salpêtrière, Paris, France; 7 Department of Clinical Neurosciences, School of Clinical Medicine, University of Cambridge, Cambridge, UK; 8 NIHR Biomedical Research Centre at Moorfields Eye Hospital and UCL Institute of Ophthalmology, London, UK

## Abstract

Wolfram syndrome (WS) is a progressive neurodegenerative disease characterized by early-onset optic atrophy and diabetes mellitus, which can be associated with more extensive central nervous system and endocrine complications. The majority of patients harbour pathogenic *WFS1* mutations, but recessive mutations in a second gene, *CISD2*, have been described in a small number of families with Wolfram syndrome type 2 (WFS2). The defining diagnostic criteria for WFS2 also consist of optic atrophy and diabetes mellitus, but unlike WFS1, this phenotypic subgroup has been associated with peptic ulcer disease and an increased bleeding tendency. Here, we report on a novel homozygous *CISD2* mutation (c.215A > G; p.Asn72Ser) in a Moroccan patient with an overlapping phenotype suggesting that Wolfram syndrome type 1 and type 2 form a continuous clinical spectrum with genetic heterogeneity. The present study provides strong evidence that this particular *CISD2* mutation disturbs cellular Ca^2+^ homeostasis with enhanced Ca^2+^ flux from the ER to mitochondria and cytosolic Ca^2+^ abnormalities in patient-derived fibroblasts. This Ca^2+^ dysregulation was associated with increased ER-mitochondria contact, a swollen ER lumen and a hyperfused mitochondrial network in the absence of overt ER stress. Although there was no marked alteration in mitochondrial bioenergetics under basal conditions, culture of patient-derived fibroblasts in glucose-free galactose medium revealed a respiratory chain defect in complexes I and II, and a trend towards decreased ATP levels. Our results provide important novel insight into the potential disease mechanisms underlying the neurodegenerative consequences of *CISD2* mutations and the subsequent development of multisystemic disease.

## Introduction

Wolfram syndrome (WS, OMIM #222300), also known historically as DIDMOAD (Diabetes Insipidus, Diabetes Mellitus, Optic Atrophy, Deafness), is an autosomal recessive disorder characterized by the association of diabetes mellitus and early-onset optic atrophy, which can occur in varying combinations with diabetes insipidus, sensorineural deafness, renal tract abnormalities or neuropsychiatric disorders (1,2). The majority of patients harbour pathogenic *WFS1* mutations, but recessive mutations in a second gene, *CISD2* (CDGSH iron-sulfur domain-containing protein 2), have been described in a few patients with Wolfram syndrome type 2 (WFS2, OMIM #604928). Unlike WFS1, patients with WFS2 have been reported to develop bleeding intestinal ulcers and defective platelet aggregation, in the absence of diabetes insipidus and psychiatric disorders (3–5).

The *CISD2* gene is located within a region on human chromosome 4q where a genetic component for human longevity has been mapped through a comparative genome analysis of centenarian siblings (6). A *Cisd2* knock-out model demonstrated that CISD2 deficiency drives premature ageing in mice and that *Cisd2* is an essential gene that regulates lifespan (7). Furthermore, an elevated level of CISD2 in transgenic mice extends the healthy lifespan of animals and delays age-associated neurodegenerative phenotypes in mice (8). *CISD2* encodes for a small protein that contains a transmembrane domain at the N-terminal and a single CDGSH domain at the C-terminal. The protein forms a homodimer harbouring two redox-active 2Fe-2S clusters (9). CISD2, which is also known as Miner 1 or ERIS, is an integral membrane protein that localizes to the mitochondria-associated ER membranes (MAMs) and the evidence so far suggests a dynamic distribution between the ER and the mitochondrial outer membrane (10). It has a role in maintaining both the structural integrity and the functional cross-talk between the ER and mitochondria, which in turn is crucial for the regulation of glucose homeostasis and insulin sensitivity (11,12). Unlike Wolframin encoded by *WFS1*, whose functions have been extensively studied, the biological functions of CISD2 still remain incompletely defined. However, both proteins seem to share overlapping functions with pivotal roles in regulating intracellular Ca^2+^ homeostasis, the ER stress response and autophagy (13–15).

Here, we report the identification of a novel homozygous *CISD2* mutation (c.215A > G; p.Asn72Ser) in a patient with a “classical” WS phenotype marked by childhood-onset insulin-dependent diabetes mellitus and progressive visual failure secondary to optic atrophy, but without peptic ulcers or defective platelet aggregation as reported previously in affected *CISD2* mutation carriers. We provide experimental evidence implicating dysregulation of Ca^2+^ homeostasis and disturbed ER-mitochondria interactions as key pathophysiological determinants that ultimately contribute to the development and progression of *CISD2*-mediated disease.

## Results

### Clinical case

**Figure 1:**
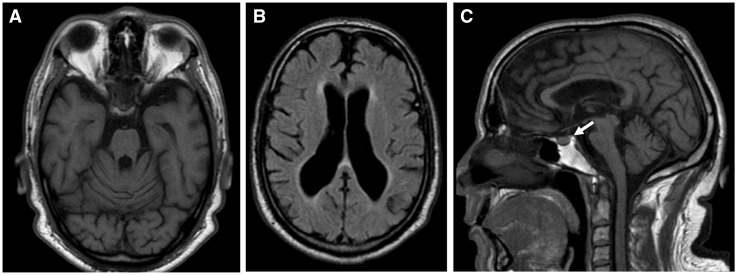
Brain magnetic resonance imaging of the proband. (**A**) Axial T1. Marked brainstem and cerebellar atrophy. (**B**) Axial T2 FLAIR. Cortical and subcortical atrophy with ventricular enlargement. (**C**) Sagittal T1. Brainstem and cerebellar atrophy, and lack of the normal posterior hypophysis hyperintense signal (arrow).

A 45-year-old Moroccan man (V3) was referred to the neurology clinic for the investigation of progressive cognitive and gait disturbances. He was born at term from consanguineous parents. His father (IV1) had late-onset type 2 diabetes mellitus and his older sister (V1) developed insulin-dependent diabetes mellitus at the age of 8 years old with severe diabetic retinopathy. At the age of 40 years old, she suffered from a cerebrovascular accident and she passed away following an acute lower limb peripheral arterial occlusion complicated by a severe infection. The proband was diagnosed with insulin-dependent diabetes mellitus at 8 years of age. He developed progressive visual impairment from the age of 33 years old, and this was initially ascribed to diabetic retinopathy. He had two episodes of hypoglycaemic coma at the age of 38 and 41 years old, and a few months after the second episode, he developed neurological impairment with progressive cognitive disturbances, apathy, gait instability, urinary incontinence and dysphagia. At his baseline assessment in the neurology clinic, neurological examination showed cerebellar ataxia, myoclonic tremor, dysarthria, dysexecutive syndrome and a pseudobulbar affect. Brain MRI showed generalised cortical and cerebellar atrophy, and bilateral optic nerve atrophy. We also observed loss of the normal hyperintense signal of the pituitary gland on T1-weighted sequence although the patient did not present with features of diabetes insipidus (Fig. 1). Ophthalmological examination with fundoscopy and optical coherence tomography (OCT) revealed bilateral optic atrophy without evidence of diabetic retinopathy. The patient’s visual acuity was 1/20 (right eye) and 1/10 (left eye). He did not report any hearing problems. The patient did not have a history of peptic ulcers or bleeding tendency and haematologic testing was unremarkable with normal platelet aggregation to ADP, collagen, ristocetin, adrenaline and arachidonic acid. An abdominal CT scan revealed several cysts in the right kidney, but none in the left kidney, liver, spleen and pancreas. There were no associated urinary tract abnormalities and renal function was preserved with normal blood levels of creatinine and urea nitrogen. Blood and tissue samples were obtained for further studies after the patient had given informed consent.

### Identification of a novel homozygous *CISD2* variant in a patient presenting with a classical wolfram syndrome phenotype

**Figure 2:**
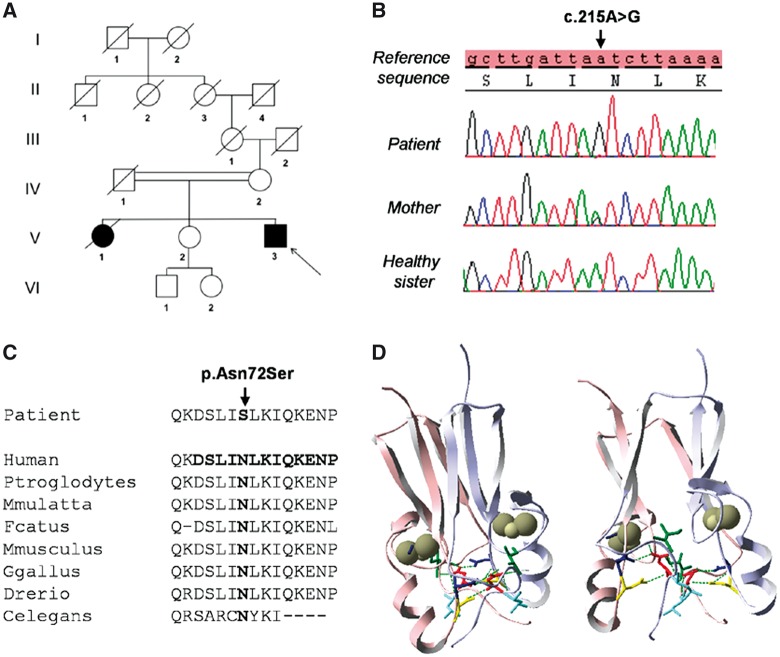
Identification of a novel *CISD2* variant (c.215A > G; p.Asn72Ser) in the proband. (**A**) Family pedigree with the solid symbols representing clinically affected individuals. (**B**) Sequence chromatograms of the proband, his mother and his healthy sister. (**C**) Cross-species protein conservation of CISD2 in the region of the altered Asparagine amino acid at position 72. (**D**) *In silico* analysis of the CISD2 homodimeric model. The individual monomers have been coloured in pink and light blue. The domain topology of the dimeric CISD2 complex consists of a six-stranded β sandwich, which forms an intertwined β cap, and a larger cluster-binding domain carrying two 2Fe-2S clusters (one per protomer) that have been highlighted as grey spheres. The Asn72 residue (shown in red) is localized in a random coil region of the cluster-binding domain and it forms potential hydrogen bonds with the neighbouring amino acids Leu73, Ile75, Asp123 and Asn124, which are represented in light blue, green, yellow and dark blue, respectively. Our *in silico* modeling indicates that the p.Asn72Ser change alters the interactions necessary for the stabilization of the cluster-binding domains and this conformational change is predicted to affect the redox and functional properties of the CISD2 protein.

The proband’s combination of childhood-onset diabetes mellitus and progressive bilateral optic atrophy complicated by more extensive neurological impairment led us to screen genes involved in WS. No mutations were found in *WFS1*, but *CISD2* sequencing revealed a novel homozygous variant, c.215A > G (p.Asn72Ser), in exon 2. His healthy mother (IV2) was heterozygous for this variant whereas his healthy sister (V2) was wild-type (Fig. 2A and B). This missense variant changes a highly-conserved asparagine (Asn) into a serine (Ser) at amino acid position 72 (Fig. 2C). It was not found in 200 ethnically and geographically matched control chromosomes and in the following SNP and exome databases: LOVD (http://lovd.euro-wabb.org/home.php?select_db=WFS1), dbSNP (http://www.ncbi.nlm.nih.gov/sites/), EVS (http://evs.gs.washington.edu/EVS/), and ExAC (http://exac.broadinstitute.org/). The c.215A > G variant was predicted to be “disease causing” based upon *in silico* analysis with SIFT (http://sift.jcvi.org/) and Mutation Taster (http://www.mutationtaster.org/). Protein modelling indicates that the Asn residue at position 72 is located within a random coil region of the cluster-binding domain. The p.Asn72Ser substitution is predicted to alter the interactions necessary for the stabilization of the cluster-binding domain, which in turn affects the redox and functional properties of the CISD2 protein (Fig. 2D).

### The c.215A > G (p.Asn72Ser) variant does not induce *CISD2* RNA mis-splicing or a reduction in CISD2 protein levels

**Figure 3:**
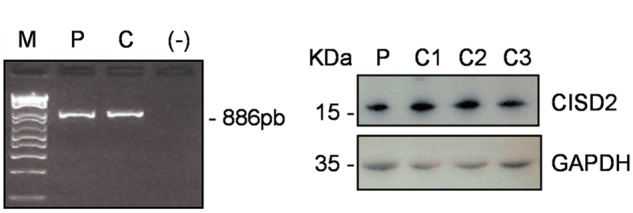
Absence of *CISD2* RNA mis-splicing and normal CISD2 protein levels in the patient’s fibroblasts. (**A**) A 886bp amplicon was obtained from cDNAs using primers in the 5’-UTR and 3’-UTR which allow the amplification of the 3 *CISD2* exons. M: molecular weight marker, P: patient, C: control; (-) negative controlc. (**B**) Western blot analysis with an anti-CISD2 antibody in fibroblasts from patient (P) and 3 control individuals (C1-C3).

Three different deleterious *CISD2* mutations have so far been reported, (c.109G > C, c.103 + 1G > A and a whole exon 2 deletion), all of which have been shown to affect mRNA splicing (3–5). We sequenced *CISD2* cDNA from the patient’s fibroblasts and quantified CISD2 protein levels by western blotting. Sequencing of the 886bp RT-PCR product did not show RNA mis-splicing and there was no decrease in CISD2 protein levels compared with control fibroblasts (Fig. 3).

### ER-mitochondrial Ca^2+^ flux is increased in patient-derived fibroblasts

**Figure 4:**
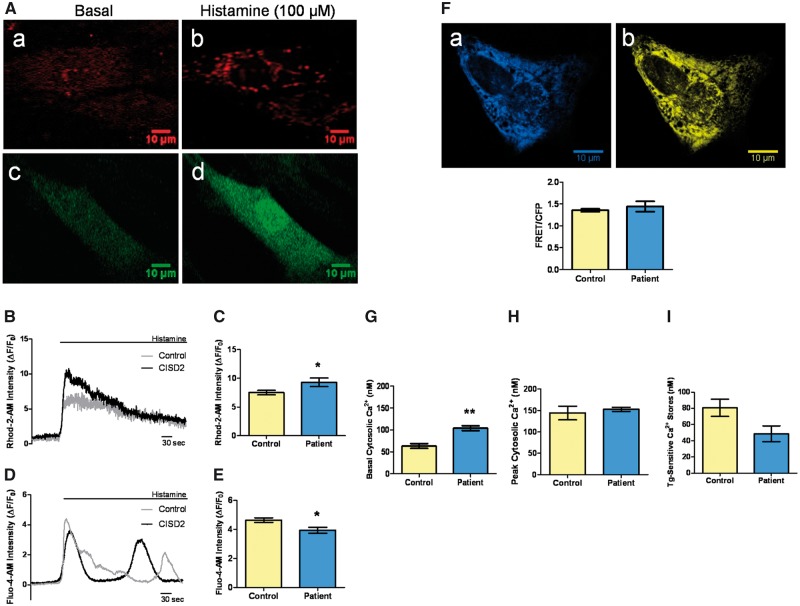
Patient’s fibroblasts showed disturbed cellular Ca^2+^ homeostasis. (**A**) Imaging of cellular Ca^2+^ flux with Ca^2+^ sensitive fluorescent dyes. Representative images demonstrating analysis of mitochondrial Ca^2+^ with the Rhod-2-AM (red) dye (a, b), and cytosolic Ca^2+^ with the Fluo-4-AM (green) dye (c, d), before and after histamine stimulation. The relevant regions of interest were analysed using the Fiji open access software. (**B**) Representative traces of mitochondrial Ca^2+^ transients. (**C**) Analysis of average peak mitochondrial Ca^2+^ following histamine stimulation. F indicates fluorescence and F_0_ indicates basal fluorescence. Data are mean ± SEM (Control *n =* 35, patient *n =*15); * *P* ≤0.05; two-tailed unpaired t test. (**D**) Representative traces of cytosolic Ca^2+^ transients. (**E**) Analysis of average peak cytosolic Ca^2+^ following histamine stimulation. Data are mean ± SEM (Control *n =* 24, patient *n =* 14); * *P* ≤ 0.05; two-tailed unpaired t test. (**F**) Imaging with the D1ER probe. Representative images of the patient’s fibroblasts expressing D1ER: CFP signal (a) and YFP signal (b). The reticular pattern is indicative of ER localization. Regions of interest were analysed using the Fiji open access software. (**G**) Increased basal cytosolic [Ca^2+^] in the patient’s fibroblasts compared with controls. Data are mean ± SEM (*n =* 3); **P ≤0.01; two-tailed unpaired *t* test. (**H**) Analysis of peak cytosolic [Ca^2+^] levels after thapsigargin-induced ER Ca^2+^ depletion. Data are mean ± SEM (*n =* 3). (**I**) Comparison of thapsigargin (Tg)-sensitive Ca^2+^ stores in the patient’s fibroblasts and controls. Data are mean ± SEM (*n =* 3).

Cellular Ca^2+^ homeostasis was investigated in patient and control fibroblasts. To assess ER-mitochondrial Ca^2+^ flux, histamine (100 µM) was used to induce IP_3_R-dependent ER Ca^2+^ release and the ensuing Ca^2+^ transients were quantified in the mitochondrial and cytosolic compartments with the Rhod-2-AM and Fluo-4-AM dyes, respectively (Fig. 4A–E). Histamine-stimulated peak mitochondrial Ca^2+^ levels were significantly increased in the patient’s fibroblasts compared with controls, in keeping with enhanced ER-mitochondrial Ca^2+^ transfer (Fig. 4C). Peak cytosolic Ca^2+^ levels were significantly decreased (Fig. 4E), but no significant differences in ER Ca^2+^ levels were observed in the patient’s fibroblasts when compared with controls (Fig. 4F). Basal cytosolic [Ca^2+^] was measured with the Fura-2-AM dye, followed by thapsigargin-induced ER Ca^2+^ depletion in the absence of extracellular Ca^2+^. Basal cytosolic [Ca^2+^] was significantly increased in *CISD2*-deficient fibroblasts (Fig. 4G), whereas peak cytosolic [Ca^2+^] following thapsigargin treatment was not significantly different when compared with controls (Fig. 4H). The thapsigargin-sensitive Ca^2+^ stores, which are defined as the difference between peak and basal cytosolic [Ca^2+^], were decreased in the patient’s fibroblasts, although this difference did not reach statistical significance (Fig. 4I).

### ER-mitochondrial contact is increased in patient-derived fibroblasts

**Figure 5:**
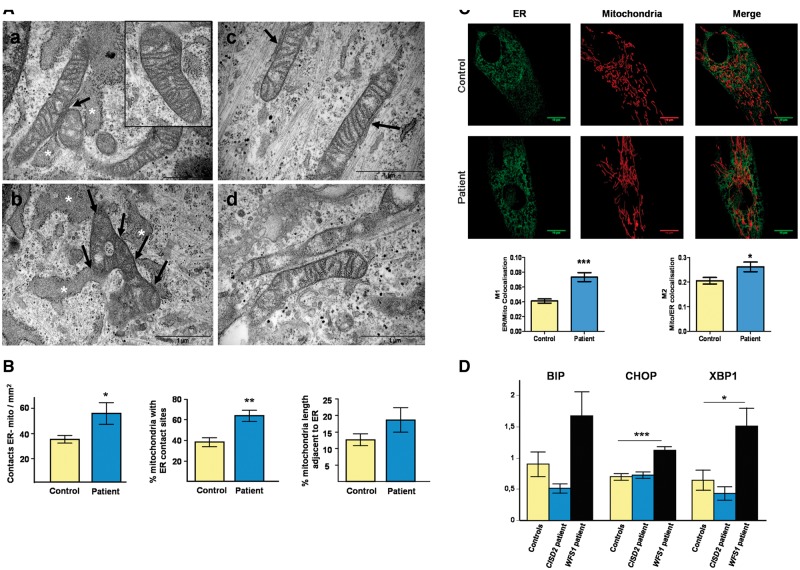
Increased ER-mitochondrial contacts observed in the patient’s fibroblasts. (**A**) Ultrastructural electron micrographs of fibroblasts from the patient (a-b) and a control individual (c-d). The contact sites between the ER and mitochondria have been indicated with arrows. The swollen ER lumen found in the patient’s cells has been highlighted with white asterisks. A higher magnification view of the typical mitochondrial morphology found in the patient’s fibroblasts has also been provided (inset of panel a). Scale bar = 1μm. (**B**) Quantification of the number of ER-mitochondria contacts expressed in mm^2^, as a percentage of mitochondria with ER contact sites, and as a percentage of the total mitochondria length adjacent to the ER normalized by mitochondrial perimeter. Data are mean ± SEM (Control *n =* 10, patient *n =* 10); ** *P* ≤0.01; * *P* ≤0.05; two-tailed unpaired t test. (**C**) Representative images of the ER and mitochondrial network (upper panels). Colocalisation analysis was performed using the Huygens Essential Analyzer and expressed as Manders’ coefficient M1 (ER/Mitochondrial (Mito) colocalisation) and M2 (Mito/ER colocalisation) to compare the patient’s fibroblasts with controls (lower panels). Data are mean ± SEM (Control *n =* 56, patient *n =* 26); *** *P* ≤0.001; * *P* ≤0.05; two-tailed unpaired t test. (**D**) qRT-PCR analysis of the ER stress markers BIP, CHOP and total XBP1. Data are mean ± SEM (*n =* 4); *** *P* ≤0.001; * *P* ≤0.05; two-tailed unpaired t test.

ER-mitochondrial contact is critical for mitochondrial Ca^2+^ uptake following ER Ca^2+^ release. Based on electron microscopy, we found a marked increase in ER apposition to mitochondria in the patient’s fibroblasts compared with controls (Fig. 5A), which was confirmed by quantitative analysis (Fig. 5B). ER-mitochondrial apposition was also assessed with live cell confocal microscopy. Fibroblasts were transfected with GFP Sec61β to visualise the ER and loaded with MitoTracker Red to label mitochondria. Z axis stacks were acquired for 3D-reconstruction. Colocalisation analysis with the Manders’ Coefficient revealed a significant increase in ER-mitochondria apposition in the patient’s fibroblasts compared with controls (Fig. 5C).

The patient’s fibroblasts also exhibited a larger lumen with a swollen appearance in some areas (Fig. 5A), indicative of possible ER stress. We therefore investigated ER stress and UPR (unfolded protein response) induction with qRT-PCR analysis of the ER stress markers BIP (an IRE1 target gene) and CHOP (a pro-apoptotic PERK target gene) in the patient’s fibroblasts, controls and fibroblasts from one patient with WFS1 secondary to confirmed recessive *WFS1* mutations. To assess activation of the ATF6 pathway, XBP1 gene expression was also assessed (Fig. 5D). There was no evidence that the *CISD2* c.215A > G (p.Asn72Ser) mutation induced significantly elevated ER stress levels unlike the WFS1 patient.

### Mitochondrial morphology and OXPHOS function are mildly affected in patient-derived fibroblasts

**Figure 6:**
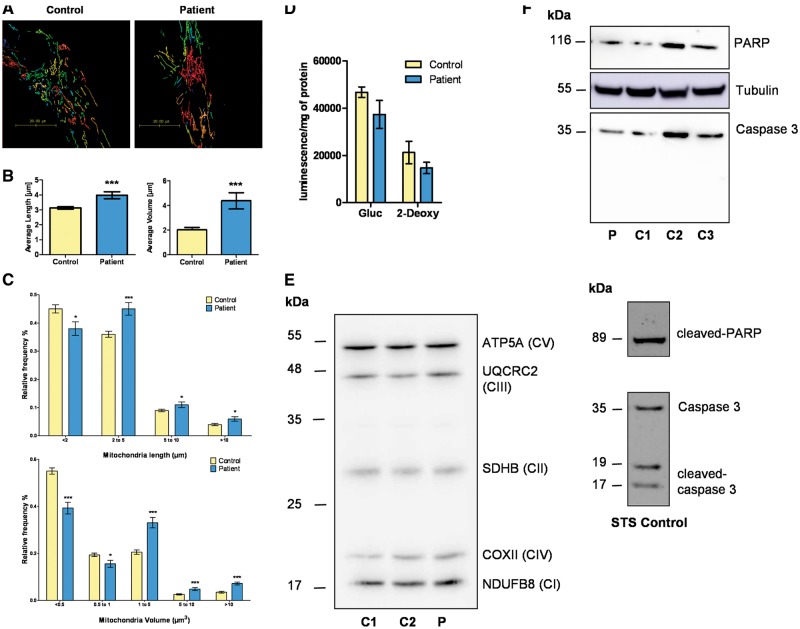
No evidence of major OXPHOS dysfunction or increased apoptosis in the patient’s fibroblasts. (**A**) Representative examples of the 3D-reconstructed mitochondrial networks in the patient’s fibroblasts and controls. Huygens Object Analyzer was used to determine the length and volume of each mitochondrial fragment. (**B**) Analysis of average mitochondrial fragment length (left panel) and volume (right panel). Data are mean ± SEM (Control *n =* 56, patient’s cells *n =* 26); *** *P* ≤0.001; two-tailed unpaired t test. (**C**) Distribution of mitochondrial fragment length (upper panel) and volume (lower panel). Data are mean ± SEM (n > 25); *** *P* ≤0.0001; * *P* ≤ 0.05; two-tailed unpaired t test. (**D**) ATP content assessed under two conditions: glucose (Gluc) and 2-deoxy-D-glucose (2-Deoxy). Data are mean ± SEM (*n =* 3); two-tailed unpaired t test. (**E**) Expression level of OXPHOS proteins. Representative western blot of ATP5A, UQCRC2, SDHB, COX II and NDUFB8 proteins performed with fibroblast lysates obtained from two control individuals (C1, C2) and the patient (P). CI-CV; complex I-V. (**F**) Representative western blots of caspase 3 and PARP in untreated controls (C1-C3) and the patient’s fibroblasts (P) (upper panel). The caspase 3 and PARP antibodies had previously been validated on an STS (staurosporin)-treated control fibroblast (lower panel).

Electron microscopy did not reveal marked ultrastructural abnormalities in the patient’s mitochondria, which demonstrated numerous, thin, well-defined cristae running perpendicularly to the mitochondrial longitudinal axis, and with a regular pattern of parallel organization (Fig. 5A). Mitochondrial network morphology was imaged and quantified with live cell confocal microscopy following Mitotracker Red staining. Interestingly, both the average length and volume of mitochondrial fragments were significantly increased in the patient’s fibroblasts compared with controls (Fig. 6A and B). Furthermore, analysis of the distribution of the length and volume of mitochondrial fragments confirmed a significant shift in mitochondrial morphology in the patient’s fibroblasts towards larger mitochondrial fragments for both length and volume, pointing towards a more fused and elongated mitochondrial network compared with controls (Fig. 6C).

**Figure 7:**
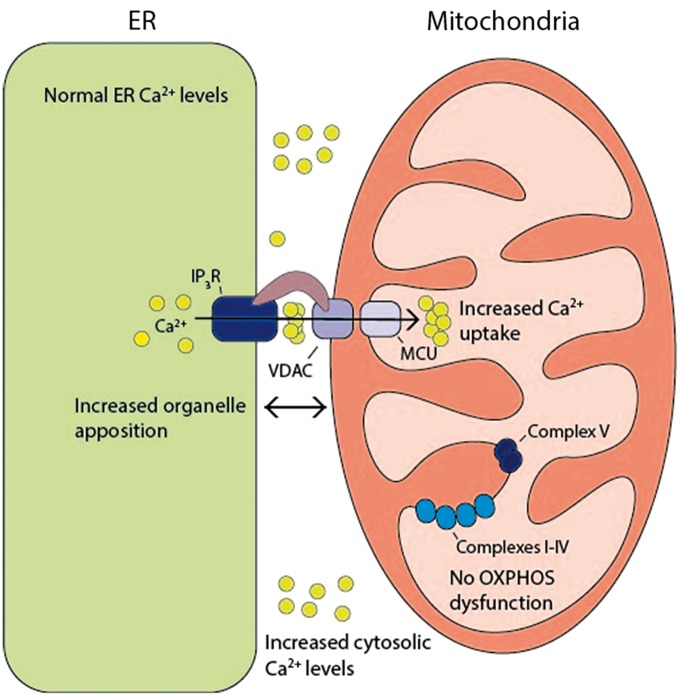
Schematic model highlighting the deleterious consequences of the *CISD2* (c.215A > G; p.Asn72Ser) mutation. This mutation was associated with disturbed Ca^2+^ homeostasis characterised by enhanced ER-mitochondrial Ca^2+^ flux and increased cytosolic [Ca^2+^]. There was increased ER-mitochondrial interorganellar contact in the absence of any major disturbance in OXPHOS dysfunction.

Spectrophotometric analysis of the patient’s fibroblasts cultivated in glucose medium revealed no respiratory chain deficiency (Table 1A). Polarographic analysis showed both normal oxygen consumption and mitochondrial substrate oxidation (Table 1C). Identical experiments were performed on fibroblasts grown in a glucose-free medium containing galactose. Galactose is a carbon source that feeds the glycolytic pathway with low efficiency and as such cells are forced to rely predominantly on OXPHOS for ATP production. Spectrophotometric analysis of the patient’s fibroblasts cultivated in galactose medium revealed a respiratory chain defect in complexes I and II (Table 1B). Polarographic analysis showed enhanced consumption of oxygen, glutamate/malate, succinate and G3P (Table 1D). There was a trend towards decreased ATP levels in the patient’s fibroblasts under both basal conditions and 2-deoxy-D-glucose (2-DG), which inhibits glycolysis and activates OXPHOS (Fig. 6D). There were no significant differences in the expression levels of key OXPHOS proteins (ATP5A, UQCRC2, SDHB, COX II and NDUFB8) in the patient’s fibroblasts compared with controls (Fig. 6E). There was no evidence of mtDNA deletions or depletion in the patient’s fibroblasts (data not shown). Western blot analysis of the apoptosis markers caspase 3 and poly (ADP-ribose) polymerase (PARP) did not indicate increased levels of apoptosis in the patient’s fibroblasts (Fig. 6F).

**Table 1:** Analysis of mitochondrial respiratory chain function in the patient’s fibroblasts. **(A,B)** Spectrophotometric analysis of mitochondrial respiratory chain enzyme activities in glucose (A) and in galactose medium (B). CS, citrate synthase. Results are expressed as extreme absolute values or absolute values for controls or patient, respectively. Values are expressed in nanomols of substrate per minute per milligram of proteins (lowered values are in grey). (**C,D)** Polarographic analysis of the respiratory chain in glucose (C) and in galactose medium (D). G3P, glycerol 3-phosphate. The results have been expressed as extreme absolute values or absolute values for controls and the patient, respectively, in nanomols of oxygen per minute per milligram of proteins

(**A**) Spectrophotometric analysis on fibroblasts cultivated in glucose medium						
Enzymatic activities	I	II	III	IV	V	CS
Control values (nmol/min/mg of proteins)	9.0–27.1	18.5–54.0	57.4–176.2	109.9–350.0	22.0–46.2	74.7–161.1
Patient	21.9	43.2	148.6	317.8	33.2	133.3

(**B**) Spectrophotometric analysis on fibroblasts cultivated in galactose medium						
Enzymatic activities	I	II	III	IV	V	CS

Control values (nmol/min/mg of proteins)	15.2–20.1	28.2–48.0	88.8–143.0	181.7–315.4	22.7–47.5	124.8–225.0
Patient	**7.5**	**24.7**	142.7	272.0	36.0	144.7

(**C**) Oxygraphic analysis on fibroblasts cultivated in glucose medium						


	Oxygen consumption			
	Intact cells	Digitonin permeabilized cells		
		Glutamate+Malate	Succinate	G3P

Control values (nmol O2/min/mg of proteins)	5.90–13.80	8.00–16.60	8.00–15.80	4.90–13.50
Patient	9.00	13.88	11.57	12.22

D Oxygraphic analysis on fibroblasts cultivated in galactose medium						


	Oxygen consumption			
	Intact cells	Digitonin permeabilized cells		
		Glutamate+Malate	Succinate	G3P

Control values (nmol O2/min/mg of proteins)	5.58 – 17.44	8.16 – 13.45	8.60 – 10.97	5.22 – 12.91
Patient	20.71	19.28	21.32	20.11

## Discussion

About 10% of patients with WS do not carry bi-allelic mutations in *WFS1* and the only other causative gene that has been identified to date in a few isolated cases worldwide is *CISD2* (16). We therefore sequenced *CISD2* in a well-defined cohort of 25 patients whose phenotypes fulfilled the diagnostic criteria for WS, but who had previously not been found to harbour pathogenic *WFS1* mutations. We identified a novel homozygous *CISD2* variant (c.215A > G; p.Asn72Ser) and the corroborative evidence we have provided all point towards its pathogenicity, namely: (i) co-segregation of this variant with affected disease status in a consanguineous family; (ii) the high degree of evolutionary conservation of the Asn amino acid residue located at position 72 in the functionally important cluster-binding domain of the protein; (iii) the absence of the c.215A > G variant in normative SNP and exome databases and; (iv) *in silico* evidence predicting a disease causing effect based on the induced conformational change of the CISD2 homodimeric protein model. Recessive *CISD2* mutations have been only described so far in five families from Jordan and Italy (3–5). Affected patients exhibited diabetes mellitus, optic atrophy and high-frequency sensorineural hearing loss. The initial reports suggested that other features of WS such as diabetes insipidus and psychiatric disorders were absent in WFS2 (3,4). However, *Rondinelli and colleagues* have now described a patient carrying a homozygous c.103 + 1 *CISD2* mutation who developed clinical and biochemical signs of diabetes insipidus (5). Of note, all the patients with *CISD2* mutations reported to date have developed peptic ulcer disease and a significant bleeding tendency, suggesting a possible genotype-phenotype association that is peculiar to WFS2. In contrast, the patient that we describe in this report with the c.215A > G missense *CISD2* variant had the classical features of Wolfram syndrome type 1, namely early-onset diabetes mellitus and progressive optic atrophy, but at the age of 45 years old, he had not been diagnosed with peptic ulcer disease or significant haematological abnormalities. His clinical course was further complicated with a cluster of neurodegenerative features such as cerebellar syndrome, myoclonic tremor, dysexecutive syndrome and a pseudobulbar affect, which have previously been reported as part of WFS1, but not WFS2 phenotypes. The patient’s brain MRI scan also revealed neuroradiological changes seen in patients with confirmed pathogenic *WFS1* mutations, namely atrophy of the optic nerve, brainstem and cerebellum, in addition to loss of the normal neurohypophyseal hypersignal (16). These similarities are probably explained, at least in part, by the substantial expression overlap of CISD2 and Wolframin in the human brain (3,17). Interestingly, the proband was found to have multiple cysts in his right kidney, which has previously not been described in the context of WS. The absence of a known family history of multicystic kidney disease, the isolated nature of the patient’s renal cysts and the clinical history are all compatible with a diagnosis of unilateral and segmental cystic disease (18), and it is likely to represent an incidental co-occurrence rather than a true causal association with the underlying *CISD2* mutation. Our data support the concept that WFS1 and WFS2 constitute a continuous clinical spectrum with overlapping phenotypes, and although *CISD2* mutations are relatively rare, this gene should be screened in patients manifesting the defining features of WS, in particular early-onset diabetes mellitus and optic atrophy, but in whom no pathogenic *WFS1* mutations have been conclusively identified.

Our experimental data indicate that the c.215A > G *CISD2* mutation disturbs cellular Ca^2+^ homeostasis characterized by enhanced Ca^2+^ flux from the ER to mitochondria and cytosolic Ca^2+^ abnormalities. CISD2 deficiency has previously been shown to disturb basal cytosolic and mitochondrial Ca^2+^ levels, albeit with conflicting results and to varying degrees in the cellular and animal models tested (11,15). No studies have assessed the impact of *CISD2* mutations on IP_3_R-dependent Ca^2+^ flux between the ER and mitochondrial compartments, which is an important potential mechanism linking ER dysfunction with mitochondrial impairment in WS (19). In the patient’s fibroblasts, Ca^2+^ flux from the ER to mitochondria was enhanced as demonstrated by significantly increased peak mitochondrial Ca^2+^ levels following IP_3_R-dependent ER Ca^2+^ release. This observation is consistent with the increased basal mitochondrial Ca^2+^ levels found *Cisd2* deficient MEFs (15). In addition, basal cytosolic [Ca^2+^] was significantly increased in the patient’s fibroblasts and this was coupled with a significant decrease in the amplitude of cytosolic Ca^2+^ transients following IP_3_R-dependent ER Ca^2+^ release. In agreement with previous studies, no difference was observed in ER Ca^2+^ levels by direct assessment with the D1ER probe (11,20). The disturbed Ca^2+^ homeostasis in the patient’s fibroblasts is therefore not due to alterations in ER Ca^2+^ levels. Interestingly, *Lu and colleagues* have described a similar increase in cytoplasmic Ca^2+^ levels in induced pluripotent stem cells (iPSC) of WS patients carrying pathogenic *WFS1* mutations, which in turn resulted in calpain activation (21). Importantly, calpain activation has been associated with the development of type 2 diabetes and the Ca2+/calpain axis is emerging as a crucial modulator of pancreatic β-cell function both in health and disease (22). Although *Lu and colleagues* did not report Ca^2+^ modification in WFS2-deficient cells, they did observe calpain hyperactivation in RNAi-mediated knockdown suggesting that it is a common molecular pathway altered in WS patients (21). It is likely that different mechanisms result in calpain hyperactivation in WS and our results suggest that *WFS2* mutations could cause calpain activation by increasing cytosolic calcium levels. Of note, increased cytosolic Ca^2+^ levels have been observed in pancreatic β-cells of patients with both type 1 and type 2 diabetes, implying that the regulation of calcium levels could prove a promising therapeutic target not only for WS, but also for other more common complex disorders characterised by ER dysfunction (23).

Several of the key folding chaperones in the ER are Ca^2+^ dependent and decreased ER Ca^2+^ content has been linked to ER stress and the induction of the UPR (15). Electron micrographs of the patient’s fibroblasts showed an expansion of the ER compartment with the lumen appearing swollen in some areas. These morphological changes have been described in cells undergoing ER stress and the UPR (24). The mRNA levels of key ER stress pathway factors were therefore quantified, but no significant increase was found compared with controls. These results are consistent with the absence of ER Ca^2+^ levels dysregulation in the patient’s fibroblasts, but contrasts with previous studies showing that *Cisd2*-deficient MEFs exhibit signs of ER stress with the pathological hallmark being an expansion of the ER compartment in order to increase the capacity to process the accumulated unfolded proteins (15,25). Similarly, although no physical ER modification was observed in pancreatic β-cells with *WFS1* mutations, these cells did exhibit signs of ER stress with an increase in CHOP expression and a strong UPR response marked by the increased activity of the three main UPR pathways, including PERK, IRE1, and ATF6 (diabetes shang 2014 923). The activation of the UPR is thought to mediate the reduced insulin content in these cells and Wolframin could act upstream of the UPR, likely in an effort to maintain ER function under protein folding stress. Further studies will be necessary to analyse the effects of Ca^2+^ homeostasis dysregulation in cell lines established from patients harbouring other *CISD2* mutations. However, it is conceivable that ER stress induces membrane expansion through UPR-mediated activation of lipid biosynthesis (26), and that the subsequent increase in ER size is sufficient on its own to dampen the stress response as a compensatory mechanism. The current body of evidence supports the hypothesis that unresolved ER stress leads to ER dysfunction, reduced processing of insulin and ultimately β-cell failure and the development of overt diabetes (27).

Strikingly, both the analysis of ER-mitochondrial signal colocalisation and electron microscopy suggested an increased degree in ER-mitochondria contact, possibly at MAM interfaces, in the patient’s fibroblasts. A central function of the MAM is the efficient transfer of Ca^2+^ from the ER to mitochondria, which is highly dependent on close apposition between these two organelles (28,29). The increased ER-mitochondria contact observed in the patient’s fibroblasts could therefore account for the increased Ca^2+^ flux between these two organelles. The increased areas of ER-mitochondrial contact is an intriguing observation and this could be due to a number of factors, including ER stress and dysregulation of the IP_3_R channel (14,15,30,31). Elevated ER stress can probably be discounted as the patient’s fibroblasts did not display increased levels of ER stress markers. CISD2 has previously been shown to interact with the IP_3_R and Bcl-2-IP_3_R interaction, in turn, negatively regulates IP_3_R-dependent Ca^2+^ transfer to mitochondria (13,32). The loss of these important regulations in the presence of a dysfunctional CISD2 protein could therefore trigger increased cytosolic [Ca^2+^] and ER-mitochondrial Ca^2+^ flux.

The transfer of Ca^2+^ from the ER to mitochondria regulates mitochondrial bioenergetics and mitochondrial-mediated apoptosis (33). Low level ER-mitochondrial Ca^2+^ transfer is necessary to maintain OXPHOS under basal conditions, but continuous mitochondrial Ca^2+^ uptake can compromise cellular bioenergetics by consuming the proton motive force (34,35). Immortalized *Cisd2* deficient MEFs were found to have increased mitochondrial Ca^2+^ loading under basal conditions, which was associated with altered mitochondrial ultrastructure, increased mitochondrial respiration and enhanced cellular ATP utilization (15). In contrast, mitochondrial respiratory chain activity was relatively preserved in the patient’s fibroblasts. OXPHOS anomalies were only unmasked when the cells were cultured in restrictive galactose medium with a defect in complexes I and II becoming apparent together with an increase in oxygen consumption, glutamate/malate, succinate and G3P. Similarly, there was a trend towards decreased ATP levels in the patient’s fibroblasts under both basal conditions and 2-DG, which inhibits glycolysis, thereby placing the cell under greater metabolic stress. Although no marked structural abnormalities were detected on electron microscopy, the patient’s fibroblasts did exhibit an elongated mitochondrial network when assessed with MitoTracker live cell staining. Enlarged and elongated mitochondria have been observed in the diaphragm muscle and myoblasts from *Cisd*2 deficient mutant mice (20). On the other hand, transient *CISD2* knockdown of less than 4 h in H1299 epithelial cells had no demonstrable effect on mitochondrial network morphology (13). Overall, these observations indicate that CISD2 is not directly involved in mitochondrial network dynamics, unlike key mediators such as the pro-fusion proteins MFN2 and OPA1 (36). Although speculative, the elongated mitochondrial network in the patient’s fibroblasts is likely to be a compensatory response, as part of the previously described phenomenon of stress induced mitochondrial hyperfusion (SIMH) (37).

In conclusion, we report a novel missense homozygous *CISD2* mutation in a patient with clinical features that differ from previously reported WFS2 case reports, emphasizing the disease spectrum of WS and the need for molecular confirmation. Although further work is needed to dissect the pathways that ultimately precipitate multisystemic neurodegeneration, the mutant CISD2 protein exerts a deleterious influence on ER-mitochondrial structure and function, providing additional lines of evidence about the central importance of interorganellar interactions in human health and disease.

## Materials and Methods

### Sequencing of *WFS1* and *CISD2*

The coding exons of *WFS1* (NM_006005.3) and *CISD2* (NM_001008388.4) were amplified with intronic primers by using standard conditions. Primers and PCR conditions are available on request. PCR products were purified with an Illustra ExoProStar enzyme (GE Healthcare, England), processed with a BidDye® Terminator Cycle Sequencing kit (Thermo Fischer, Foster City, CA, USA) and analyzed on an ABI 3130 XL automated sequencer (Applied Biosystems).

### RT-PCR analysis

Total RNA was extracted from fibroblasts by using the Trizol reagent according to the manufacturer’s instructions and treated by DNAse I (Invitrogen, Carlsbad, CA). cDNA synthesis was performed using the Kit Transcriptor first strand cDNA Synthesis (Roche, USA). The 3 exons of *CISD2* were sequenced using primers in the 5’UTR (5’-AGCTTGGCCAGAGCGGA-3’) and 3’UTR (5’-AACCAAATGCAGTTTGGAAGG-3’). Measuring ER stress was performed by qRT-PCR using TaqMan Gene Expression Assays (ThermoFisher, USA): XBP1 (Hs00231936_m1), CHOP (Hs0035 8796_g1), BIP (Hs00607129_gH) and HPRT (Hs99999909_m1). qRT-PCR reactions were run in duplicate on a LightCycler 480 (Roche, USA) and assays were repeated four times. Fibroblasts from 2 healthy subjects and one WS patient were used as controls. The results were analysed by students *t*-test.

### OXPHOS spectrophotometric measurements

Enzymatic spectrophotometric measurements of the OXPHOS respiratory chain complexes and citrate synthase were performed at 37 °C on patient’s fibroblasts according to standard procedures (38). Proteins were measured according to Bradford microassay (39).

### Polarographic study

Polarographic studies on fibroblasts of intact cell respiration and digitonin (0.004%) permeabilized cells mitochondrial substrate oxidation were carried out as previously described (38). Proteins were measured according to Bradford microassay (39).

### Western blotting

50 µg of total protein extracts, obtained as previously described (40), were separated on an acrylamide gel by SDS-PAGE and transferred to a PVDF membrane (Millipore, Saint-Quentin, France). Specific proteins were detected by using rabbit polyclonal anti-CISD2 (1/1000, Pierce#PA5-34545) and anti-GAPDH (1/20000, Abcam #ab9485). A cocktail of anti-human total OXPHOS complex antibodies (Mitosciences, Eugene, USA) was used at 1/1000. Anti-mouse or anti-rabbit secondary antibody (Dako) was used at 1/5000 and signals were detected using a chemiluminescence system (Immobilon Western HRP Chemilumiscent substrates, Millipore).

### Mitochondrial DNA analysis

Total DNA was extracted using standard phenol chloroform extraction procedure. Mitochondrial DNA quantification in fibroblasts was performed by real-time quantitative PCR adapted from the method described by Sarzi *et al.* (41). Primers and conditions are available on request.

### Cell culture

Primary fibroblast cultures were established using standard procedures in RPMI supplemented with 10% Fetal Bovine Serum, 45μg/ml uridine and 275μg/ml sodium pyruvate. Cultures were incubated at 37 °C with 5% CO2. For galactose conditions, medium was replaced 24h before experiments by glucose-free medium containing 5 mM galactose and 5 mM pyruvate (42).

### *In silico* analysis of the homodimeric CISD2 structure

The 2.1 Å coordinate set for the C-terminal water-soluble domain of Miner 1 (pdb code: 3fnv; residues 57-135) was downloaded. Swiss-Pdb Viewer 3.7 software was used to generate the ribbon diagram of the homodimeric CISD2 complex and analyze the structural and conformational disruptions induced by the c.215A > G (p.Asn72Ser) *CISD2* mutation.

### Electron microscopy

For ultrastructural analysis, cells were fixed in 1.6% glutaraldehyde in 0.1 M phosphate buffer, rinsed in 0.1 M cacodylate buffer, post-fixed for 1h in 1% osmium tetroxide and 1% potassium ferrocyanide in 0.1 M cacodylate buffer to enhance the staining of membranes. Cells were rinsed in distilled water, dehydrated in alcohols and lastly embedded in epoxy resin. Contrasted ultrathin sections (70 nm) were analyzed under a JEOL 1400 transmission electron microscope mounted with a Morada Olympus CCD camera.

### Ca^2+^ measurements

Fibroblast cell lines were seeded at 100,000 cells/dish on glass bottom dishes (Willco, HBS-3522). Fluo-4-am (ThermoFisher) and Rhod-2-am (ThermoFisher) were used to evaluate cytosolic and mitochondrial Ca^2+^, respectively. 4 µM Fluo-4-am was loaded at 37 °C for 15 min or 8 µM Rhod-2-am was loaded at room temperature for 30 min. Cells were washed twice with PBS and incubated in Tyrode’s solution (137 mM NaCl, 2.7 mM KCl, 1 mM MgCl_2_, 0.2 mM Na_2_HPO_4_, 12 mM NaHCO_3_, 2 mM CaCl_2_, 10 mM Glucose, pH 7) for 15 min. Dynamic measurements of cytosolic and mitochondrial Ca^2+^ were performed at room temperature with a Nikon A1R inverted confocal microscope equipped with a x60, 1.40 NA oil objective in resonant scanning mode at 3.7fps for 5 min. After 1 min (providing basal fluorescence) histamine (100 µM) was added to evoke IP_3_R dependent ER Ca^2+^ release and the Ca^2+^ transient was monitored. Image analysis was performed in Fiji, the background was subtracted and Ca^2+^ levels were calculated as (F-F_o_)/F_o_ where F indicates fluorescence and F_o_ indicates basal fluorescence.

The ER targeted fluorescence resonance energy transfer (FRET) based probe D1ER was used to measure ER Ca^2+^ levels. Cells were transiently transfected with D1ER ∼48 h before imaging in opti-MEM (ThermoFisher) with GeneJuice Transfection Reagent (Merck Millipore) and 2 µg of plasmid DNA (pcDNA-D1ER was a gift from Amy Palmer & Roger Tsien). Cells were washed twice with PBS and imaged in HBSS without phenol red or Ca^2+^ and supplemented with 5 mM glucose and 25 mM HEPES (Sigma-Aldrich) at pH 7.4. Live cell imaging was performed at room temperature with a Nikon A1R inverted confocal microscope equipped with a x60, 1.40 NA oil objective. The filters used were a 457 nm excitation filter and two emission filters: 482 nm for CFP and 540 nm for YFP. The FRET to CFP ratio was calculated by ratioing the emission at 540 nm and 482 nm.

Cytosolic Ca^2+^ levels were determined using the ratiometric Ca^2+^ sensitive fluorescent dye Fura-2-am at basal and following thapsigargin induced ER Ca^2+^ depletion. Fibroblast cell lines were seeded at 40,000 cells/well on black 96 well optical bottom microplates (ThermoFisher) the day before the experiment. Cells were loaded with 5µM Fura-2 (ThermoFisher) at 37 °C for 1 h 30 min, then washed twice with PBS and incubated for a further 30 min. Fluorescence was monitored on a Varioskan^TM^ LUX multimode microplate reader at emission wavelength 510 nm following alternate excitations at 340 nm and 380 nm in HBSS without phenol red or Ca^2+^. ER Ca^2+^ depletion was triggered by the addition of 1 µm thapsigargin. To calibrate the Fura-2 probe, R_min_ was determined by the addition of 2 µM ionomycin (ThermoFisher) and 3 mM EGTA (Sigma-Aldrich), and R_max_ was determined by the addition of 2 µM ionomycin (ThermoFisher) and 10 mM CaCl_2_ (Sigma-Aldrich). The standard equation was used to calculate cytosolic [Ca^2+^]: [Ca^2+^] = K_d_ x Q x [(R-R_min_)/R_max_-R], where R is the 340/380 ratio, R_min_ is the 340/380 ratio under Ca^2+^ free conditions, R_max_ is the 340/380 ratio under Ca^2+^ saturating conditions, Q is the ratio of emission intensity at 380 nm for Ca^2+^ free and Ca^2+^ saturated Fura-2 and Kd was assumed to be 225 nM.

### Mitochondria–ER interactions and morphological analysis

Fibroblast cell lines were seeded at 100,000 cells/dish on glass bottom dishes. GFP-Sec61β was transiently transfected to detect the ER. Cells were transfected ∼24 h before imaging in opti-MEM (ThermoFisher) with GeneJuice Transfection Reagent (Merck Millipore) and 1 µg of plasmid DNA. Mitotracker red (75 nM, ThermoFisher) was loaded at 37 °C for 30 min to detect mitochondria. Cells were washed twice with PBS and imaged in MEM without phenol red (ThermoFisher) supplemented with 25 mM HEPES (Sigma-Aldrich). Live cells were imaged at room temperature with a Nikon A1R inverted confocal microscope equipped with a x60, 1.40 NA oil objective. Sixty nine Z-stacks were acquired across the cell at 0.11 µM increments using a high speed piezo Z stage. All image analysis were performed in Huygens Essential Software (SVI). Images were deconvolved and a 3D-reconstructed. Colocalisation of the ER and mitochondria signals was quantified using the Manders’ coefficient. ER total volume and the length and volume of each mitochondrial fragment was analysed in Huygens Object Analyser.

### ATP measurements

ATP levels were measured using the CellTiter-Glo Luminescent Assay (Promega) according to the manufacturer’s instructions. Fibroblast cell lines were seeded at 40,000 cells/well on 96 well white microplates (Thermo Scientific). The cells were incubated for 1 h 30 min at 37 °C in either glucose (5 mM, Sigma-Aldrich) or, 2-Deoxy-D-glucose (5 mM, Sigma-Aldrich). Following incubation, 90 µL buffer was removed and 100 µL of CellTiter-glo reagent was added to each well. Following 25 min incubation in the dark with shaking, luminescence was measured on a Luminoskan Ascent (Thermo Scientific) with a 1000 ms integration time. Luminescence signal was normalized to mg of protein using the Bradford assay.
