# A novel *CISD2* mutation associated with a classical Wolfram syndrome phenotype alters Ca^2+^ homeostasis and ER-mitochondria interactions

**DOI:** 10.1093/hmg/ddx130

**Published:** 2017-04-20

**Authors:** Cécile Rouzier, David Moore, Cécile Delorme, Sandra Lacas-Gervais, Samira Ait-El-Mkadem, Konstantina Fragaki, Florence Burté, Valérie Serre, Sylvie Bannwarth, Annabelle Chaussenot, Martin Catala, Patrick Yu-Wai-Man, Véronique Paquis-Flucklinger

**Affiliations:** 1 Université Côte d’Azur, CHU, Inserm, CNRS, IRCAN, France; 2 Wellcome Trust Centre for Mitochondrial Research, Institute of Genetic Medicine, International Centre for Life, Newcastle University, Newcastle upon Tyne NE1 3BZ, UK; 3 Fédération de Neurologie, Université Pierre et Marie Curie et Groupe Hospitalier Pitié-Salpêtrière, Paris, France; 4 Joint Centre for Applied Electron Microscopy, Nice Sophia-Antipolis University, Nice, France; 5 UMR7592 CNRS, Jacques Monod Institute, Paris Diderot University, Paris, France; 6 UMR 7622 CNRS et UPMC et Fédération de Neurologie, Université Pierre et Marie Curie et Groupe Hospitalier Pitié-Salpêtrière, Paris, France; 7 Department of Clinical Neurosciences, School of Clinical Medicine, University of Cambridge, Cambridge, UK; 8 NIHR Biomedical Research Centre at Moorfields Eye Hospital and UCL Institute of Ophthalmology, London, UK

*Human Molecular Genetics* 2017, doi: 10.1093/hmg/ddx060

The Authors would like to apologize for missing information in their acknowledgement. The full acknowledgement should read as follows:

We thank Didier Dormant (MD, PhD) for performing the cerebral MRI of the patient and Georges Challe (MD) for the ophthalmological examination. We thank Cécile Delettre from the Institute of Neurosciences (Montpellier, France) for providing fibroblasts from the WFS1 patient. We are also grateful to Alain Lacampagne and Jeremy Fauconnier (Montpellier, France) for their help with the calcium experiments. We thank Gaëlle Augé, Bernadette Chafino and Charlotte Cochaud for technical help.

This has now been corrected in the original paper and online.

